# COVID-19 coronavirus vaccine design using reverse vaccinology and machine learning

**DOI:** 10.1101/2020.03.20.000141

**Published:** 2020-03-21

**Authors:** Edison Ong, Mei U Wong, Anthony Huffman, Yongqun He

**Affiliations:** 1 Department of Computational Medicine and Bioinformatics, University of Michigan, Ann Arbor, MI 48109, USA; 2 Unit for Laboratory Animal Medicine, Department of Microbiology and Immunology, University of Michigan, Ann Arbor, MI 48109, USA

## Abstract

To ultimately combat the emerging COVID-19 pandemic, it is desired to develop an effective and safe vaccine against this highly contagious disease caused by the SARS-CoV-2 coronavirus. Our literature and clinical trial survey showed that the whole virus, as well as the spike (S) protein, nucleocapsid (N) protein, and membrane protein, have been tested for vaccine development against SARS and MERS. We further used the Vaxign reverse vaccinology tool and the newly developed Vaxign-ML machine learning tool to predict COVID-19 vaccine candidates. The N protein was found to be conserved in the more pathogenic strains (SARS/MERS/COVID-19), but not in the other human coronaviruses that mostly cause mild symptoms. By investigating the entire proteome of SARS-CoV-2, six proteins, including the S protein and five non-structural proteins (nsp3, 3CL-pro, and nsp8–10) were predicted to be adhesins, which are crucial to the viral adhering and host invasion. The S, nsp3, and nsp8 proteins were also predicted by Vaxign-ML to induce high protective antigenicity. Besides the commonly used S protein, the nsp3 protein has not been tested in any coronavirus vaccine studies and was selected for further investigation. The nsp3 was found to be more conserved among SARS-CoV-2, SARS-CoV, and MERS-CoV than among 15 coronaviruses infecting human and other animals. The protein was also predicted to contain promiscuous MHC-I and MHC-II T-cell epitopes, and linear B-cell epitopes localized in specific locations and functional domains of the protein. Our predicted vaccine targets provide new strategies for effective and safe COVID-19 vaccine development.

## Introduction

The emerging Coronavirus Disease 2019 (COVID-19) pandemic poses a massive crisis to global public health. As of March 11, 2020, there were 118,326 confirmed cases and 4,292 deaths, according to the World Health Organization (WHO), and WHO declared the COVID-19 as a pandemic on the same day. The causative agent of the COVID-19 disease is the severe acute respiratory syndrome coronavirus 2 (SARS-CoV-2). Coronaviruses can cause animal diseases such as avian infectious bronchitis caused by the infectious bronchitis virus (IBV), and pig transmissible gastroenteritis caused by a porcine coronavirus^1^. Bats are commonly regarded as the natural reservoir of coronaviruses, which can be transmitted to humans and other animals after genetic mutations. There are seven known human coronaviruses, including the novel SARS-CoV-2. Four of them (HCoV-HKU1, HCoV-OC43, HCoV-229E, and HCoV-NL63) have been circulating in the human population worldwide and cause mild symptoms^2^. Coronavirus became prominence after Severe acute respiratory syndrome (SARS) and Middle East Respiratory Syndrome (MERS) outbreaks. In 2003, the SARS disease caused by the SARS-associated coronavirus (SARS-CoV) infected over 8,000 people worldwide and was contained in the summer of 2003^3^. SARS-CoV-2 and SARS-CoV share high sequence identity^4^. The MERS disease infected more than 2,000 people, which is caused by the MERS-associated coronavirus (MERS-CoV) and was first reported in Saudi Arabia and spread to several other countries since 2012^5^.

There is no human vaccine on the market to prevent COVID-19, and there is an urgent need to develop a safe and effective vaccine to prevent this highly infectious disease. Coronaviruses are positively-stranded RNA viruses with its genome packed inside the nucleocapsid (N) protein and enveloped by the membrane (M) protein, envelope (E) protein, and the spike (S) protein^6^. While many coronavirus vaccine studies targeting different structural proteins were conducted, most of these efforts eventually ceased soon after the outbreak of SARS and MERS. With the recent COVID-19 pandemic outbreak, it is urgent to resume the coronavirus vaccine research. As the immediate response to the ongoing pandemic, the first testing in humans of the mRNA-based vaccine targeting the S protein of SARS-CoV-2 (ClinicalTrials.gov Identifier: NCT04283461, Table 1) started on March 16, 2020. As the most superficial and protrusive protein of the coronaviruses, S protein plays a crucial role in mediating virus entry. In the SARS vaccine development, the full-length S protein and its S1 subunit (which contains receptor binding domain) have been frequently used as the vaccine antigens due to their ability to induce neutralizing antibodies that prevent host cell entry and infection. However, studies showed that S protein-based vaccination did not provide full protection and sometimes raise safety concerns^7,8^. In the meantime, many other research groups and companies are also putting great efforts into developing and manufacture COVID-19 vaccines.

In recent years, the development of vaccine design has been revolutionized by the reverse vaccinology (RV), which aims to first identify promising vaccine candidate through bioinformatics analysis of the pathogen genome. RV has been successfully applied to vaccine discovery for pathogens such as Group B meningococcus and led to the license Bexsero vaccine^9^. Among current RV prediction tools^10,11^, Vaxign is the first web-based RV program^12^ and has been used to successfully predict vaccine candidates against different bacterial and viral pathogens^13–15^. Recently we have also developed a machine learning approach called Vaxign-ML to enhance prediction accuracy^16^.

In this study, we first surveyed the existing coronavirus vaccine development status, and then applied the Vaxign RV and Vaxign-ML approaches to predict COVID-19 protein candidates for vaccine development. We identified six possible adhesins, including the structural S protein and five other non-structural proteins, and three of them (S, nsp3, and nsp8 proteins) were predicted to induce high protective immunity. The S protein was predicted to have the highest protective antigenicity score and it has been extensively studied as the target of coronavirus vaccines by other researchers. Here we selected nsp3 protein as an alternative vaccine candidate, which was predicted to have the second-highest protective antigenicity score yet, has not been considered in any vaccine studies. We investigated the sequence conservation and immunogenicity of the multi-domain nsp3 protein as a vaccine candidate.

## Results

### Published research and clinical trial coronavirus vaccine studies

To better understand the current status of coronavirus vaccine development, we systematically surveyed the development of vaccines for coronavirus from the ClinicalTrials.gov database and PubMed literature (as of March 17, 2020). Extensive effort has been made to develop a safe and effective vaccine against SARS or MERS, and the most advance clinical trial study is currently at phase II (Table 1). It is a challenging task to quickly develop a safe and effective vaccine for the on-going COVID-19 pandemic.

There are two primary design strategies for coronavirus vaccine development: the usage of the whole virus or genetically engineered vaccine antigens that can be delivered through different formats. The whole virus vaccines include inactivated^17^ or live attenuated vaccines^18,19^ (Table 2). The two live attenuated SARS vaccines mutated the exoribonuclease and envelop protein to reduce the virulence and/or replication capability of the SARS-CoV. Overall, the whole virus vaccines can induce a strong immune response and protect against coronavirus infections. Genetically engineered vaccines that target specific coronavirus protein are often used to improve vaccine safety and efficacy. The coronavirus antigens such as S protein, N protein, and M protein can be delivered as recombinant DNA vaccine and viral vector vaccine (Table 2).

### N protein is conserved among SARS-CoV-2, SARS-CoV, and MERS-CoV, but missing from the other four human coronaviruses causing mild symptoms

We first used the Vaxign analysis framework^12,16^ to compare the full proteomes of seven human coronavirus strains (SARS-CoV-2, SARS-CoV, MERS-CoV, HCoV-229E, HCoV-OC43, HCoV-NL63, and HCoV-HKU1). The proteins of SARS-CoV-2 were used as the seed for the pan-genomic comparative analysis. The Vaxign pan-genomic analysis reported only the N protein in SARS-CoV-2 having high sequence similarity among the more severe form of coronavirus (SARS-CoV and MERS-CoV), while having low sequence similarity among the more typically mild HCoV-229E, HCoV-OC43, HCoV-NL63, and HCoV-HKU1. The sequence conservation suggested the potential of N protein as a candidate for the cross-protective vaccine against SARS and MERS. The N protein was also evaluated and used for vaccine development (Table 2). The N protein packs the coronavirus RNA to form the helical nucleocapsid in virion assembly. This protein is more conserved than the S protein and was reported to induce an immune response and neutralize coronavirus infections^20^. However, a study also showed the linkage between N protein and severe pneumonia or other serious liver failures related to the pathogenesis of SARS^21^.

### Six adhesive proteins in SARS-CoV-2 identified as potential vaccine targets

The Vaxign RV analysis predicted six SARS-CoV-2 proteins (S protein, nsp3, 3CL-PRO, and nsp8–10) as adhesive proteins (Table 3). Adhesin plays a critical role in the virus adhering to the host cell and facilitating the virus entry to the host cell^22^, which has a significant association with the vaccine-induced protection^23^. In SARS-CoV-2, S protein was predicted to be adhesin, matching its primary role in virus entry. The structure of SARS-CoV-2 S protein was determined^24^ and reported to contribute to the host cell entry by interacting with the angiotensin-converting enzyme 2 (ACE2)^25^. Besides S protein, the other five predicted adhesive proteins were all non-structural proteins. In particular, nsp3 is the largest non-structural protein of SARS-CoV-2 comprises various functional domains^26^.

### Three adhesin proteins were predicted to induce strong protective immunity

The Vaxign-ML pipeline computed the protegenicity (protective antigenicity) score and predicted the induction of protective immunity by a vaccine candidate^16^. The training data consisted of viral protective antigens, which were tested to be protective in at least one animal challenge model^27^. The performance of the Vaxign-ML models was evaluated (Table S1 and Figure S1), and the best performing model had a weighted F1-score of 0.94. Using the optimized Vaxign-ML model, we predicted three proteins (S protein, nsp3, and nsp8) as vaccine candidates with significant protegenicity scores (Table 3). The S protein was predicted to have the highest protegenicity score, which is consistent with the experimental observations reported in the literature. The nsp3 protein is the second most promising vaccine candidate besides S protein. There was currently no study of nsp3 as a vaccine target. The structure and functions of this protein have various roles in coronavirus infection, including replication and pathogenesis (immune evasion and virus survival) ^26^. Therefore, we selected nsp3 for further investigation, as described below.

### Nsp3 as a vaccine candidate

**Figure 1.**
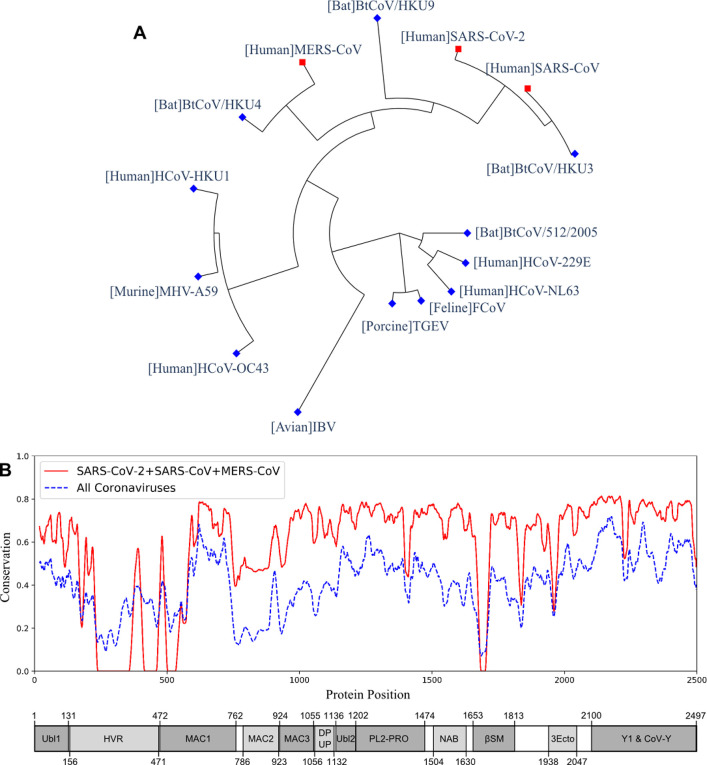
The phylogeny and sequence conservation of coronavirus nsp3. (A) Phylogeny of 15 strains based on the nsp3 protein sequence alignment and phylogeny analysis. (B) The conservation of nsp3 among different coronavirus strains. The red line represents the conservation among the four strains (SARS-CoV, SARS-CoV-2, MERS, and BtCoV-HKU3). The blue line was generated using all the 15 strains. The bottom part represents the nsp3 peptides and their sizes. The phylogenetically close four strains have more conserved nsp3 sequences than all the strains being considered.

The multiple sequence alignment and the resulting phylogeny of nsp3 protein showed that this protein in SARS-CoV-2 was more closely related to the human coronaviruses SARS-CoV and MERS-CoV, and bat coronaviruses BtCoV/HKU3, BtCoV/HKU4, and BtCoV/HKU9. We studied the genetic conservation of nsp3 protein (Figure 1A) in seven human coronaviruses and eight coronaviruses infecting other animals (Table S2). The five human coronaviruses, SARS-CoV-2, SARS-CoV, MERS-CoV, HCoV-HKU1, and HCoV-OC43, belong to the beta-coronavirus while HCoV-229E and HCoV-NL63 belong to the alpha-coronavirus. The HCoV-HKU1 and HCoV-OC43, as the human coronavirus with mild symptoms clustered together with murine MHV-A59. The more severe form of human coronavirus SARS-CoV-2, SARS-CoV, and MERS-CoV grouped with three bat coronaviruses BtCoV/HKU3, BtCoV/HKU4, and BtCoV/HKU9.

When evaluating the amino acid conservations relative to the functional domains in nsp3, all protein domains, except the hypervariable region (HVR), macro-domain 1 (MAC1) and beta-coronavirus-specific marker βSM, showed higher conservation in SARS-CoV-2, SARS-CoV, and MERS-CoV (Figure 1B). The amino acid conservation between the major human coronavirus (SARS-CoV-2, SARS-CoV, and MERS-CoV) was plotted and compared to all 15 coronaviruses used to generate the phylogenetic of nsp3 protein (Figure 1B). The SARS-CoV domains were also plotted (Figure 1B), with the relative position in the multiple sequence alignment (MSA) of all 15 coronaviruses (Table S3 and Figure S2).

**Figure 2.**
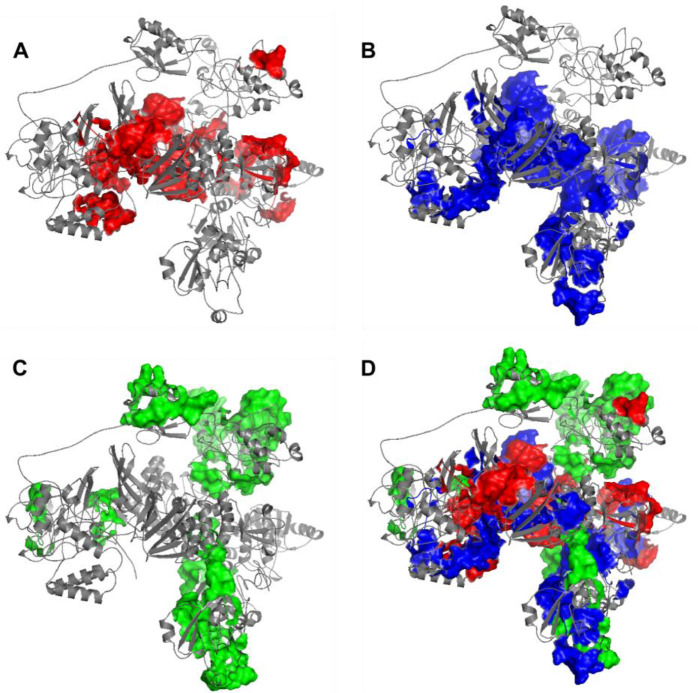
Predicted 3D structure of nsp3 protein highlighted with (A) MHC-I T cell epitopes (red), (B) MHC-II (blue) T cell epitopes, (C) linear B cell epitopes (green), and the merged epitopes. MHC-I epitopes are more internalized, MHC-II epitopes are more mixed, and B cells are more shown on the surface.

**Figure 3.**
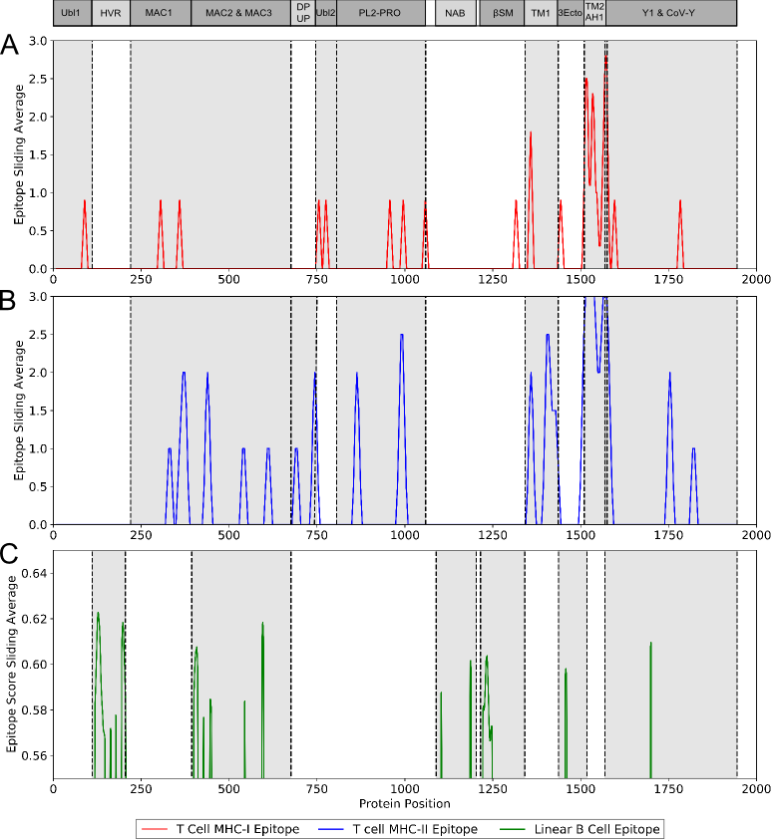
Immunogenic region of nsp3 between SARS-CoV-2 and the four conservation strains. (A) MHC-I (red) T cell epitope (B) MHC-II (blue) T cell epitope (C) linear B cell epitope (green).

The immunogenicity of nsp3 protein in terms of T cell MHC-I & MHC-II and linear B cell epitopes was also investigated. There were 28 and 42 promiscuous epitopes predicted to bind the reference MHC-I & MHC-II alleles, which covered the majority of the world population, respectively (Table S4–5). In terms of linear B cell epitopes, there were 14 epitopes with BepiPred scores over 0.55 and had at least ten amino acids in length (Table S6). The 3D structure of SARS-CoV-2 protein was plotted and highlighted with the T cell MHC-I & MHC-II, and linear B cell epitopes (Figure 2). The predicted B cell epitopes were more likely located in the distal region of the nsp3 protein structure. Most of the predicted MHC-I & MHC-II epitopes were embedded inside the protein. The sliding averages of T cell MHC-I & MHC-II and linear B cell epitopes were plotted with respect to the tentative SARS-CoV-2 nsp3 protein domains using SARS-CoV nsp3 protein as a reference (Figure 3). The ubiquitin-like domain 1 and 2 (Ubl1 and Ubl2) only predicted to have MHC-I epitopes. The Domain Preceding Ubl2 and PL2-PRO (DPUP) domain had only predicted MHC-II epitopes. The PL2-PRO contained both predicted MHC-I and MHC-II epitopes, but not B cell epitopes. In particular, the TM1, TM2, and AH1 were predicted helical regions with high T cell MHC-I and MHC-II epitopes^28^. The TM1 and TM2 are transmembrane regions passing the endoplasmic reticulum (ER) membrane. The HVR, MAC2, MAC3, nucleic-acid binding domain (NAB), βSM, Nsp3 ectodomain; (3Ecto), Y1, and CoV-Y domain contained predicted B cell epitopes. Finally, the Vaxign RV framework also predicted 2 regions (position 251–260 and 329–337) in the MAC1 domain of nsp3 domain having high sequence similarity to the human mono-ADP-ribosyltransferase PARP14 (NP_060024.2).

## Discussion

Our prediction of the potential SARS-CoV-2 antigens, which could induce protective immunity, provides a timely analysis for the vaccine development against COVID-19. Currently, most coronavirus vaccine studies use the whole inactivated or attenuated virus, or target the structural proteins such as the spike (S) protein, nucleocapsid (N) protein, and membrane (M) protein (Table 2). But the inactivated or attenuated whole virus vaccine might induce strong adverse events. On the other hand, vaccines targeting the structural proteins induce a strong immune response^20,29,30^. In some studies, these structural proteins, including the S and N proteins, were reported to associate with the pathogenesis of coronavirus^21,31^ and might raise safety concern. A study has shown increased liver pathology in the vaccinated ferrets immunized with modified vaccinia Ankara-S recombinant vaccine^32^. Although there were no other adverse events reported in other animal studies, the safety and efficacy of these vaccination strategies has not been tested in human clinical trials. Our study applied the state-of-the-art Vaxign reserve vaccinology (RV) and Vaxign-ML machine learning strategies to the entire SARS-CoV-2 proteomes including both structural and non-structural proteins for vaccine candidate prediction. Our results indicate for the first time that many non-structural proteins could be used as potential vaccine candidates.

The SARS-CoV-2 S protein was identified by our Vaxign and Vaxign-ML analysis as the most favorable vaccine candidate. First, the Vaxign RV framework predicted the S protein as a likely adhesin, which is consistent with the role of S protein for the invasion of host cells. Second, our Vaxign-ML predicted that the S protein had a high protective antigenicity score. These results confirmed the role of S protein as the important target of COVID-19 vaccines. However, the S protein exists in many coronaviruses, and many non-pathogenic human coronaviruses also use S protein to cell invasion. For example, despite markedly weak pathogenicity, HCoV-NL63 also uses S protein and employs the angiotensin-converting enzyme 2 (ACE2) for cellular entry^33^. This suggests that the S protein is not the only factor determining the infection level of a human coronavirus. In addition, targeting only the S protein may induce high serum-neutralizing antibody titers but cannot induce sufficient protective efficacy^34^. Thus, alternative vaccine antigens may be considered.

The SARS-CoV-2 nsp3 protein was predicted to be a potential vaccine candidate, as shown by its predicted second-highest protective antigenicity score, adhesin property, promiscuous MHC-I & MHC-II T cell epitopes, and B cell epitopes. The nsp3 is the largest non-structural protein that includes multiple functional domains to support viral pathogenesis^26^. The multiple sequence alignment of nsp3 also showed higher sequence conservation in most of the functional domains in SARS-CoV-2, SARS-CoV, and MERS-CoV, than in all 15 coronavirus strains (Fig. 1B). The induction of nsp3-specific immunity would likely help the host to fight against the infection. Besides the S and nsp3 proteins, our study also suggested four additional vaccine candidates, including 3CL-pro, nsp8, nsp9, and nsp10. All these proteins were predicted as adhesins, and the nsp8 protein was also predicted to have a significant protective antigenicity score.

Our predicted non-structural proteins (nasp3, 3CL-pro, nsp8, nsp9, and nsp10) are not part of the viral structural particle, and none of the non-structural proteins have been evaluated as vaccine candidates. The SARS/MERS/COVID-19 vaccine studies so far target the structural (S/M/N) proteins. Still, the non-structural proteins have been used effective vaccine antigens to stimulate protective immunity against many viruses. For example, the non-structural protein NS1 was found to induce protective immunity against the infections by flaviviruses^35^. The non-structural proteins of the hepatitis C virus were reported to induce HCV-specific vigorous and broad-spectrum T-cell responses^36^. The non-structural HIV-1 gene products were also shown to be valuable targets for prophylactic or therapeutic vaccines^37^. Therefore, it is reasonable to consider the SARS-CoV-2 non-structural proteins as possible vaccine targets, as suggested by the present study.

Instead of using a single protein as the vaccine antigen, we would like to propose the development of a “cocktail vaccine” as an effective strategy for COVID-19 vaccine development. A typical cocktail vaccine includes more than one antigen to cover different aspects of protection^39,40^. The licensed Group B meningococcus Bexsero vaccine, which was developed via reverse vaccinology, contains three protein antigens^9^. To develop an efficient and safe COVID-19 cocktail vaccine, it is possible to mix the structural (e.g., S protein) and non-structural (e.g., nsp3) viral proteins. The other proteins identified in our study may also be considered as possible vaccine targets. The benefit of a cocktail vaccine strategy could induce immunity that can protect the host against not only the S-ACE2 interaction and viral entry to the host cells, but also protect against the accessary non-structural adhesin proteins (e.g., nsp3), which might also be vital to the viral entry and replication. The usage of more than one antigen allows us to reduce the volume of each antigen and thus reducing the induction of adverse events. Nonetheless, the potentials of these predicted non-structural protein targets in vaccine development need to be experimentally validated.

For rational COVID-19 vaccine development, it is critical to understand the fundamental host-coronavirus interaction and protective immune mechanism^7^. Such understanding may not only provide us guidance in terms of antigen selection but also facilitate our design of vaccine formulations. For example, an important foundation of our prediction in this study is based on our understanding of the critical role of adhesin as a virulence factor as well as protective antigen. The choice of DNA vaccine, recombinant vaccine vector, and another method of vaccine formulation is also deeply rooted in our understanding of pathogen-specific immune response induction. Different experimental conditions may also affect results^41,42^. Therefore, it is crucial to understand the underlying molecular and cellular mechanisms for rational vaccine development.

## Methods

### Annotation of literature and database records.

We annotated peer-reviewed journal articles stored in the PubMed database and the ClinicalTrials.gov database. From the peer-reviewed articles, we identified and annotated those coronavirus vaccine candidates that were experimentally studied and found to induce protective neutralizing antibody or provided immunity against virulent pathogen challenge.

### Vaxign prediction.

The SARS-CoV-2 sequence was obtained from NCBI. All the proteins of six known human coronavirus strains, including SARS-CoV, MERS-CoV, HCoV-229E, HCoV-OC43, HCoV-NL63, and HCoV-HKU1 were extracted from Uniprot proteomes^43^. The full proteomes of these seven coronaviruses were then analyzed using the Vaxign reverse vaccinology pipeline^12,16^. The Vaxign program predicted serval biological features, including adhesin probability^44^, transmembrane helix^45^, orthologous proteins^46^, and protein functionss^12,16^.

### Vaxign-ML prediction.

The ML-based RV prediction model was build following a similar methodology described in the Vaxign-ML^16^. Specifically, the positive samples in the training data included 397 bacterial and 178 viral protective antigens (PAgs) recorded in the Protegen database^27^ after removing homologous proteins with over 30% sequence identity. There were 4,979 negative samples extracted from the corresponding pathogens’ Uniprot proteomes^43^ with sequence dis-similarity to the PAgs, as described in previous studies^47–49^. Homologous proteins in the negative samples were also removed. The proteins in the resulting dataset were annotated with biological and physicochemical features. The biological features included adhesin probability^44^, transmembrane helix^45^, and immunogenicity^50^. The physicochemical features included the compositions, transitions and distributions^51^, quasi-sequence-order^52^, Moreau-Broto autocorrelation^53,54^ and Geary auto-correlation^55^ of various physicochemical properties such as charge, hydrophobicity, polarity, and solvent accessibility^56^. Five supervised ML classification algorithms, including logistic regression, support vector machine, k-nearest neighbor, random forest ^57^, and extreme gradient boosting (XGB) ^58^ were trained on the annotated proteins dataset. The performance of these models was evaluated using a nested five-fold cross-validation (N5CV) based on the area under receiver operating characteristic curve, precision, recall, weighted F1-score, and Matthew’s correlation coefficient. The best performing XGB model was selected to predict the protegenicity score of all SARS-CoV-2 isolate Wuhan-Hu-1 (GenBank ID: MN908947.3) proteins, downloaded from NCBI. A protein with protegenicity score over 0.9 is considered as strong vaccine immunity induction (weighted F1-score > 0.94 in N5CV).

### Phylogenetic analysis.

The protein nsp3 was selected for further investigation. The nsp3 proteins of 14 coronaviruses besides SARS-CoV-2 were downloaded from the Uniprot (Table S2). Multiple sequence alignment of these nsp3 proteins was performed using MUSCLE^59^ and visualized via SEAVIEW^60^. The phylogenetic tree was constructed using PhyML^61^, and the amino acid conservation was estimated by the Jensen-Shannon Divergence (JSD)^62^. The JSD score was also used to generate a sequence conservation line using the nsp3 protein sequences from 4 or 13 coronaviruses.

### Immunogenicity analysis.

The immunogenicity of the nsp3 protein was evaluated by the prediction of T cell MHC-I and MHC-II, and linear B cell epitopes. For T cell MHC-I epitopes, the IEDB consensus method was used to predicting promiscuous epitopes binding to 4 out of 27 MHC-I reference alleles with consensus percentile ranking less than 1.0 score^50^. For T cell MHC-II epitopes, the IEDB consensus method was used to predicting promiscuous epitopes binding to more than half of the 27 MHC-II reference alleles with consensus percentile ranking less than 10.0. The MHC-I and MHC-II reference alleles covered a wide range of human genetic variation representing the majority of the world population^63,64^. The linear B cell epitopes were predicted using the BepiPred 2.0 with a cutoff of 0.55 score^65^. Linear B cell epitopes with at least ten amino acids were mapped to the predicted 3D structure of SARS-CoV-2 nsp3 protein visualized via PyMol^66^. The predicted count of T cell MHC-I and MHC-II epitopes, and the predicted score of linear B cell epitopes were computed as the sliding averages with a window size of ten amino acids. The nsp3 protein 3D structure was predicted using C-I-Tasser^67^ available in the Zhang Lab webserver (https://zhanglab.ccmb.med.umich.edu/C-I-TASSER/2019-nCov/).

## Supplementary Material

Supplement 1

Supplement 2

## Figures and Tables

**Table 1.** Reported SARS-CoV, MERS-CoV, SARS-CoV-2 vaccine clinical trials.

Virus	Location	Phase	Year	Identifier	Vaccine Type

SARS-CoV	United States	I	2004	NCT00099463	recombinant DNA vaccine (S protein)
SARS-CoV	United States	I	2007	NCT00533741	whole virus vaccine
SARS-CoV	United States	I	2011	NCT01376765	recombinant protein vaccine (S protein)
MERS	United Kingdom	I	2018	NCT03399578	vector vaccine (S protein)
MERS	Germany	I	2018	NCT03615911	vector vaccine (S protein)
MERS	Saudi Arabia	I	2019	NCT04170829	vector vaccine (S protein)
MERS	Germany, Netherland	I	2019	NCT04119440	vector vaccine (S protein)
MERS	Russia	I,II	2019	NCT04128059	vector vaccine (protein not specified)
MERS	Russia	I,II	2019	NCT04130594	vector vaccine (protein not specified)
SARS-CoV2	United States	I	2020	NCT04283461	mRNA-based vaccine (S protein)

**Table 2.** Vaccines tested for SARS-CoV and MERS-CoV.

Vaccine name	Vaccine type	Antigen	PMID

**SARS vaccines**			
CTLA4-S DNA vaccine	DNA	S	15993989
*Salmonella*-CTLA4-S DNA vaccine	DNA	S	15993989
*Salmonella*-tPA-S DNA vaccine	DNA	S	15993989
Recombinant spike polypeptide vaccine	Recombinant	S	15993989
N protein DNA vaccine	DNA	N	15582659
M protein DNA vaccine	DNA	M	16423399
N protein DNA vaccine	DNA	N	16423399
N+M protein DNA vaccine	DNA	N, M	16423399
tPA-S DNA vaccine	DNA	S	15993989
β-propiolactone-inactivated SARS-CoV vaccine	Inactivated virus	whole virus	16476986
MA-ExoN vaccine	Live attenuated	MA-ExoN	23142821
rMA15-ΔE vaccine	Live attenuated	MA15	23576515
Ad S/N vaccine	Viral vector	S,N	16476986
ADS-MVA vaccine	Viral vector	S	15708987
MVA/S vaccine	Viral vector	S	15096611
**MERS vaccines**			
England1 S DNA Vaccine	DNA	S	26218507
MERS-CoV pcDNA3.1-S1 DNA vaccine	DNA	S	28314561
Inactivated whole MERS-CoV (IV) vaccine	Inactivated virus	whole virus	29618723
England1 S DNA +England1 S protein subunit Vaccine	Mixed	S1	26218507
England1 S1 protein subunit Vaccine	Subunit	S1	26218507
MERS-CoV S vaccine	Subunit	S	29618723
rNTD vaccine	Subunit	NTD of S	28536429
rRBD vaccine	Subunit	RBD of S	28536429
Ad5.MERS-S vaccine	Viral vector	S	25192975
Ad5.MERS-S1 vaccine	Viral vector	S1 subunit	25192975
VSVΔG-MERS vaccine	Viral vector	S	29246504

Abbreviation: S, surface glycoprotein; N, nucleocapsid phosphoprotein; M, membrane glycoprotein; Exon, exoribonuclease; NTD, N-terminal domain; RBD, receptor binding domain.

**Table 3.** Vaxign-ML Prediction and adhesin probability of all SARS-CoV-2 proteins.

		Protein	Vaxign-ML Score	Adhesin Probability

orf1ab	nsp1	Host translation inhibitor	79.312	0.297
	nsp2	Non-structural protein 2	89.647	0.319
	nsp3	Non-structural protein 3	**95.283** *	**0.524** ^#^
	nsp4	Non-structural protein 4	89.647	0.289
	3CL-PRO	Proteinase 3CL-PRO	89.647	**0.653** ^#^
	nsp6	Non-structural protein 6	89.017	0.320
	nsp7	Non-structural protein 7	89.647	0.269
	nsp8	Non-structural protein 8	**90.349** *	**0.764** ^#^
	nsp9	Non-structural protein 9	89.647	**0.796** ^#^
	nsp10	Non-structural protein 10	89.647	**0.769** ^#^
	RdRp	RNA-directed RNA polymerase	89.647	0.229
	Hel	Helicase	89.647	0.398
	ExoN	Guanine-N7 methyltransferase	89.629	0.183
	NendoU	Uridylate-specific endoribonuclease	89.647	0.254
	2’-O-MT	2’-O-methyltransferase	89.647	0.421
S		Surface glycoprotein	**97.623** *	**0.635** ^#^
ORF3a		ORF3a	66.925	0.383
E		envelope protein	23.839	0.234
M		membrane glycoprotein	84.102	0.282
ORF6		ORF6	33.165	0.095
ORF7		ORF7a	11.199	0.451
ORF8		ORF8	31.023	0.311
N		nucleocapsid phosphoprotein	89.647	0.373
ORF10		ORF10	6.266	0.0

* denotes Vaxign-ML predicted vaccine candidate.

# denotes predicted adhesin.
